# Euphorine

**Published:** 1891-02-07

**Authors:** 


### THERAPEUTICS.

### EUPHORINE.

This is the name given by Professor Giacosa to phenylurethan, a body obtained by the action of chlorocarbonic acid upon aniline. It is a white crystalline powder with a slight aromatic odour, and a taste which, at first scarcely perceptible, becomes sharper after a little time, somewhat resembling that of cloves. It is not very soluble in water, but freely so in alcohol, even very dilute, as in white wine. It is antipyretic, antiseptic, and, in some cases, analgesic, and useful also in rheumatism. The antipyretic action begins in about an hour or less, and lasts for five to seven hours. Collapse has never yet been observed, even when the temperature has fallen below the normal. The doses are about half those of antipyrin, and generally in febrile conditions 15 to 20 grains may be given in the day without any unpleasant results; but it is as well not to give more than 1½ grain at a time at first. In acute rheumatism the daily dosage should be from 20 to 30 grains, and the single doses as large as can be borne. In chronic ophthalmia and in indolent ulcers Giacosa has found the best results from using it as a fine powder dusted over the surface, which soon becomes red, the pus disappearing, and granulation proceeding rapidly.

